# Cellular plasticity: a double-edged sword driving malignant transformation and adaptive remodeling in stem cells

**DOI:** 10.3389/fcell.2026.1752590

**Published:** 2026-01-30

**Authors:** Xue Du, Zhimei Lin, Yijun Zeng, Chunbao Chen

**Affiliations:** 1 Department of Oncology and Hematology, The 3RD Affiliated Hospital of Chengdu Medical College, Chengdu Pidu District People’s Hospital, Chengdu, China; 2 Department of Neurosurgery, The 3RD Affiliated Hospital of Chengdu Medical College, Chengdu Pidu District People’s Hospital, Chengdu, China

**Keywords:** adaptive remodeling, cellular plasticity, malignant transformation, microenvironment response, stem cells

## Abstract

This review systematically examines the dual roles of cellular plasticity in stem cell biology. Stem cells leverage their inherent plasticity to achieve adaptive tissue remodeling, including migration, differentiation, and microenvironment interactions during development, regeneration, and homeostasis maintenance. However, this property can also be hijacked by cancer cells, particularly cancer stem cells, as a key mechanism for malignant transformation, invasion, metastasis, and therapeutic resistance. This review will delve into the molecular foundations of cellular plasticity, such as cytoskeletal dynamics, nuclear plasticity, and cell junctional plasticity, analyzing the regulatory similarities and differences between physiological remodeling and pathological transformation. Finally, we will explore the potential and challenges of targeting cellular plasticity as a novel anti-cancer strategy.

## 1 Introduction

Stem cells have emerged as a research hotspot in the life sciences due to their unique biological properties (Choudhery and Umezawa, 2025). In recent years, with the continuous advancement of cell biology research, the regulatory role of cellular physical properties in cell function has gained increasing attention, with cellular plasticity being particularly crucial (Huber et al., 2015). In developmental biology and cancer biology, plasticity typically refers to transcriptional plasticity, epigenetic plasticity, or cellular fate plasticity, describing the ability of cells to switch their lineage identity or differentiation state (Flavahan et al., 2017). In contrast, this review primarily focuses on mechanical cellular plasticity, which refers to the ability of cells to undergo deformation in response to external forces. The magnitude of this deformation is determined by the composition and arrangement of the cytoskeleton, the mechanical properties of the cell membrane, and the regulation of intracellular signaling pathways (Paluch et al., 2016). This encompasses the capacity of cells to undergo reversible or irreversible deformation in response to external mechanical forces.

Under physiological conditions, appropriate cellular plasticity ensures stem cells can successfully complete proliferation, differentiation, migration, and colonization processes to meet the requirements of the body (Scarpa and Mayor, 2016). Under pathological conditions, abnormal changes in cellular plasticity may induce malignant transformation of stem cells, leading to tumorigenesis and progression (Northcott et al., 2018). Concurrently, when the microenvironment surrounding stem cells undergoes alterations, such as hypoxia, nutrient deprivation, or changes in mechanical stress, cellular plasticity adapts through adaptive adjustments. This facilitates the remodeling of biological functions to sustain stem cell survival (Semenza, 2012; Engler et al., 2006).

Given the extensive applications of stem cells in tissue repair and regenerative medicine, their potential safety concerns, particularly the risk of malignant transformation, have gradually emerged as a critical challenge that cannot be overlooked during clinical translation (Ben-David and Benvenisty, 2011). Research indicates that stem cells undergoing prolonged *in vitro* expansion, gene editing, or exposure to abnormal microenvironmental stimuli may develop genomic instability, epigenetic abnormalities, and signaling pathway dysregulation, thereby increasing the risk of tumorigenesis (Mimeault and Batra, 2009). In hematological malignancy research, abnormal hematopoietic stem/progenitor cells are considered the critical cellular basis for leukemia initiation and maintenance. Targeted interventions against their specific molecular markers or key signaling pathways may offer novel strategies to enhance therapeutic efficacy and reduce recurrence rates (Kikushige and Miyamoto, 2015). In solid tumors, targeting pathways associated with tumor stem cells holds promise for overcoming the limitations of conventional therapies (Batlle and Clevers, 2017). Therefore, delving into the role and mechanisms of cellular plasticity in the malignant transformation and adaptive remodeling of stem cells is crucial for elucidating the pathogenesis of stem cell-related diseases and developing novel therapeutic strategies.

## 2 Regulatory mechanisms of cellular plasticity

**FIGURE 1:**
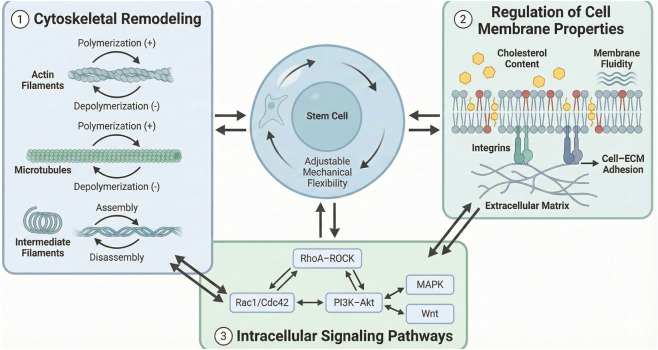
Regulatory mechanisms of cellular plasticity in stem cells. The central stem cell displays adjustable mechanical flexibility. Three major regulatory modules surround the cell. Panel 1: Cytoskeletal remodeling, including actin filaments, microtubules, and intermediate filaments with polymerization and depolymerization states. Panel 2: Regulation of cell membrane properties, showing lipid bilayer composition, cholesterol content, membrane fluidity, integrins, and cell–extracellular matrix adhesion. Panel 3: Intracellular signaling pathways, including RhoA–ROCK, Rac1/Cdc42, PI3K–Akt, MAPK, and Wnt signaling. Arrows indicate bidirectional regulation between signaling pathways, cytoskeleton, and membrane mechanics.

The dynamic equilibrium of cellular plasticity underpins the normal functioning of stem cells. Its regulation involves a multifactorial, multilevel synergistic process, primarily encompassing three core pathways: cytoskeletal remodeling, modulation of cell membrane properties, and activation of intracellular signaling pathways (Figure 1).

### 2.1 Cytoskeletal remodeling

The cytoskeleton serves as the core structure maintaining cell morphology and determining cellular plasticity, primarily composed of microfilaments, microtubules, and intermediate filaments. The dynamic changes in these three components directly influence cellular plasticity (Fletcher and Mullins, 2010). The polymerization and depolymerization of microfilaments are key regulators of cellular plasticity. Enhanced actin polymerization forming dense fiber networks increases cellular rigidity and reduces flexibility, whereas actin depolymerization enhances cellular plasticity (Hotulainen and Lappalainen, 2006; McGrath et al., 2000). During stem cell differentiation, osteogenic-differentiating stem cells exhibit elevated actin polymerization and reduced cellular plasticity to adapt to the rigid microenvironment of bone tissue. Conversely, adipogenic-differentiating stem cells demonstrate actin depolymerization and enhanced cellular plasticity (McBeath et al., 2004; Tan et al., 2014).

Microtubules, as a crucial component of the cytoskeleton, also influence cellular plasticity through their stability (Brangwynne et al., 2006). Microtubule assembly and disassembly are regulated by microtubule-associated proteins. Enhanced microtubule stability increases a cell’s resistance to deformation, reducing its flexibility, while microtubule depolymerization renders cells more susceptible to deformation (Desai and Mitchison, 1997). Although intermediate filaments do not directly participate in rapid cellular deformation, they form connective networks with microfilaments and microtubules, thereby enhancing the stability of the cellular structure and indirectly regulating the range of cellular plasticity.

### 2.2 Regulation of cell membrane properties

As the barrier connecting cells to their external environment, the mechanical properties of the cell membrane, such as elasticity and fluidity are closely linked to cellular plasticity (Diz-Muñoz et al., 2013). The primary components of the cell membrane are the lipid bilayer and membrane proteins. Changes in the composition and structure of these components directly influence membrane properties, thereby altering cellular plasticity.

From the perspective of the lipid bilayer, the degree of saturation and chain length of fatty acids are key influencing factors. Higher saturated fatty acid content and longer chain lengths result in tighter lipid molecule packing, reduced membrane fluidity, and consequently diminished cellular plasticity (Lingwood and Simons, 2010). Conversely, increased unsaturated fatty acid content enhances membrane fluidity and increases cellular plasticity. Furthermore, cholesterol content within the cell membrane also regulates membrane properties. Moderate cholesterol levels increase membrane rigidity, while excessive cholesterol disrupts membrane structural stability, leading to abnormal cellular plasticity (Maxfield and van Meer, 2010).

Membrane proteins such as integrins and adhesion molecules transmit external signals into cells by binding to the cytoskeleton and extracellular matrix, while also influencing the mechanical state of the cell membrane (Hynes, 2002). Following binding to the extracellular matrix, integrins activate intracellular signaling pathways that promote actin polymerization, indirectly reducing cellular plasticity. Phosphorylation modifications of membrane proteins may alter their binding capacity to the cytoskeleton, thereby regulating cellular plasticity (Parsons et al., 2010).

### 2.3 Activation of intracellular signaling pathways

Multiple intracellular signaling pathways indirectly achieve precise regulation of cellular plasticity by modulating cytoskeletal remodeling and cell membrane properties. Among these, the Rho family GTPase signaling pathway and the PI3K-Akt signaling pathway are particularly crucial (Jaffe and Hall, 2005; Fruman et al., 2017).

Rho family GTPases, including RhoA, Rac1, and Cdc42, serve as core regulators of cytoskeletal remodeling (Heasman and Ridley, 2008). RhoA promotes actin polymerization and contraction by activating the downstream effector molecule Rho-associated protein kinase (ROCK), thereby increasing cellular rigidity. Rac1 and Cdc42 primarily influence cellular extension and deformation capacity by regulating actin branching, thereby modulating cellular plasticity (Amano et al., 2010). When stem cells encounter external mechanical stimuli, Rac1 activation promotes actin branching network formation, enhancing cellular plasticity to adapt to the stimulus signal.

The PI3K-Akt signaling pathway regulates the expression and modification of cytoskeletal proteins. Upon activation, Akt phosphorylates actin-binding proteins, inhibiting actin depolymerization and increasing cellular rigidity (Shamsan et al., 2024). Concurrently, Akt modulates the synthesis of membrane lipids, altering membrane fluidity and indirectly influencing cellular plasticity (Porstmann et al., 2008). Furthermore, pathways such as MAPK signaling and Wnt signaling can interact with the aforementioned pathways to collectively maintain the dynamic equilibrium of cellular plasticity (Mendoza et al., 2011).

## 3 The role of cellular plasticity in stem cell malignant transformation

**FIGURE 2:**
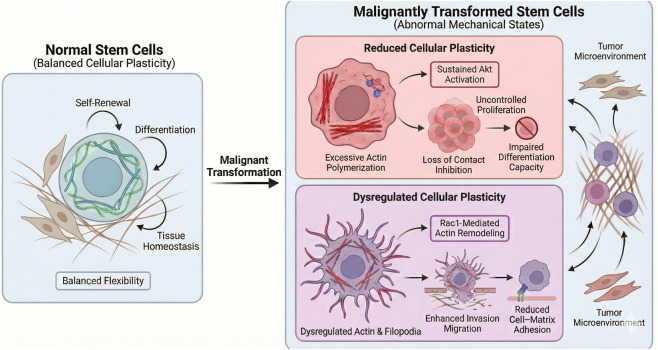
Abnormal cellular plasticity drives stem cell malignant transformation. The figure shows a transition from normal stem cells with balanced cellular plasticity to malignantly transformed stem cells with abnormal mechanical states. Reduced cellular plasticity is associated with excessive actin polymerization, sustained Akt activation, uncontrolled proliferation, loss of contact inhibition, and impaired differentiation capacity. Dysregulated cellular flexibility promotes enhanced invasion and migration through Rac1-mediated actin remodeling, filopodia formation, and reduced cell–matrix adhesion.

Stem cell malignant transformation is a complex, multi-stage process involving multiple factors, characterized by features such as uncontrolled proliferation, loss of differentiation capacity, and enhanced invasive migration abilities. Abnormal changes in cellular plasticity serve as a key driver of stem cell malignant transformation, propelling the process by regulating the physiological behavior of stem cells (Wei et al., 2015) (Figure 2).

### 3.1 Abnormal cellular plasticity and uncontrolled stem cell proliferation

Under normal conditions, stem cell proliferation is tightly regulated, with cellular plasticity maintained at an optimal level to ensure a balance between proliferation and differentiation (Vining and Mooney, 2017). When cellular plasticity abnormally decreases, this equilibrium is disrupted, triggering uncontrolled stem cell proliferation. Mechanistically, reduced cellular plasticity is often accompanied by enhanced actin polymerization. A dense actin network activates proliferation-related signaling pathways within the cell (Aragona et al., 2013), mechanosensitive co-activators such as (Yes-associated protein (YAP) and transcriptional coactivator with PDZ-binding motif (YAP/TAZ) (Zanconato et al., 2016). During actin cytoskeletal stiffening, YAP/TAZ undergoes dephosphorylation and translocates to the nucleus, driving the expression of cyclins and cell proliferation-related genes, thereby promoting cell proliferation (Mukherjee et al., 2025).

In models of malignant transformation of bone marrow mesenchymal stem cells, decreased cellular plasticity leads to sustained phosphorylation of Akt, which in turn promotes the expression of cell cycle regulatory proteins. This enables stem cells to overcome proliferation restrictions and enter a state of uncontrolled proliferation (Tan et al., 2014). In the early stages of malignant transformation, enhanced intracellular actin polymerization and stress fiber formation lead to reduced overall cellular flexibility and increased rigidity (Paszek et al., 2005). By enhancing downstream phosphoinositide 3-kinase (PI3K) signaling through integrin and focal adhesion complexes, PI3K catalyzes the generation of phosphatidylinositol-3,4,5-trisphosphate (PIP3), providing a membrane-localized platform for protein kinase B (PKB/Akt) activation (Schwartz and Ginsberg, 2002; Schlaepfer and Hunter, 1998). Dense actin networks activate YAP/TAZ, which upon entering the nucleus can directly upregulate the gene expression of multiple positive regulators in the Akt pathway, including PI3K subunits and growth factor receptors (Dupont et al., 2011). Once continuously phosphorylated, Akt acts as a central signaling node, systematically downregulating cell cycle inhibitors while simultaneously upregulating pro-proliferative proteins (Hemmings and Restuccia, 2012).

Reduced cellular plasticity creates a self-perpetuating vicious cycle. Rigid cytoskeletal structures trigger sustained Akt signaling, which in turn promotes cell proliferation and cytoskeletal remodeling, further diminishing plasticity and intensifying Akt signaling. This enables cells to evade dependence on normal growth factors and ignore contact inhibition signals. Additionally, abnormal cellular plasticity impairs the contact inhibition function of stem cells. During growth, normal stem cells suppress proliferation through intercellular adhesion signals when cell density reaches a certain threshold, a process known as contact inhibition. When cellular plasticity decreases, adhesion molecules on the cell membrane malfunction, failing to effectively transmit contact inhibition signals. This leads to sustained stem cell proliferation, laying the groundwork for malignant transformation (Gupta and Chaudhuri, 2022; Bijonowski et al., 2025).

### 3.2 Abnormal cellular plasticity and loss of stem cell differentiation capacity

Loss of differentiation capacity is one of the core characteristics of malignant transformation in stem cells, and alterations in cellular plasticity directly impact the differentiation potential of stem cells. Under normal conditions, stem cells adjust their flexibility in response to microenvironmental signals to initiate corresponding differentiation programs. As previously described, stem cells differentiating into bone tissue reduce their flexibility, while those differentiating into adipose tissue increase their flexibility (Engler et al., 2006; Gilbert et al., 2010). When cellular plasticity becomes abnormal, this “flexibility-differentiation” matching relationship is disrupted, leading to the loss of stem cell differentiation capacity.

In stem cells undergoing malignant transformation, a significant reduction in cellular plasticity is commonly observed, and this decrease correlates positively with the loss of the stem cells multipotent differentiation capacity (Suresh, 2007). Taking neural stem cells as an example, normal neural stem cells can differentiate into various cell types such as neurons and astrocytes in response to microenvironmental signals. In contrast, malignant transformed neural stem cells, such as neuroblastoma stem cells, exhibit markedly reduced flexibility and can only undergo disordered proliferation, failing to complete normal differentiation processes (Pugh et al., 2013). Abnormal mechanical signals conveyed by reduced cellular plasticity can jointly lock the stem cell self-renewal transcriptional program by inhibiting classical differentiation pathways like Wnt and Notch (Maurice and Angers, 2025; Giniger, 2012) and abnormally activating the YAP/TAZ mechanosensitive pathway, ultimately leading to differentiation arrest (Azzolin et al., 2014).

### 3.3 Abnormal cellular plasticity and enhanced invasive migration capacity of stem cells

Enhanced invasive and migratory capacity serves as a critical prerequisite for tumor metastasis following malignant transformation of stem cells, while alterations in cellular plasticity represent a core factor regulating stem cell invasion and migration capabilities (Vird et al., 2022). Compared to normal stem cells, malignantly transformed stem cells typically exhibit enhanced abnormal flexibility. Some solid tumor stem cells demonstrate reduced flexibility but can still achieve invasion and migration through cytoskeletal remodeling. This flexibility change enables them to more readily penetrate the extracellular matrix, breach tissue barriers, and complete the invasion and migration process.

When stem cellular plasticity increases, the fluidity of the cell membrane rises, making cells more prone to deformation and enabling them to pass smoothly through narrow gaps in the extracellular matrix (Liu et al., 2015). Simultaneously, enhanced flexibility promotes the activation of the Rac1 signaling pathway, regulating the formation of branched pseudopodia rich in actin filaments, thereby providing propulsion for cell migration (Ridley, 2015). In tumor stem cell research, malignant transformed tumor stem cells exhibit significantly higher flexibility than normal tissue stem cells. Through Rac1-mediated actin remodeling, they form abundant filopodia, enhancing their invasive capacity into surrounding tissues and thereby promoting tumor metastasis (Wei et al., 2015; Xu et al., 2012). Furthermore, abnormal cellular plasticity impairs stem cell adhesion to the extracellular matrix. By reducing cell-matrix attachment strength, it facilitates detachment from the primary tumor site and promotes distant metastasis (Paluch et al., 2016).

## 4 Effects of cellular plasticity on adaptive remodeling in stem cells

**FIGURE 3:**
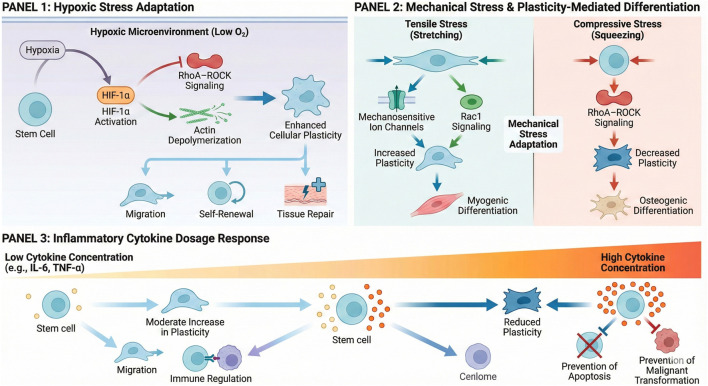
Cellular plasticity mediates adaptive remodeling of stem cells under microenvironmental stress. Panel 1: Hypoxic microenvironment activates HIF-1α signaling, inhibits RhoA–ROCK, promotes actin depolymerization, and enhances cellular plasticity to support migration, self-renewal, and tissue repair. Panel 2: Mechanical stress adaptation, where tensile stress activates mechanosensitive ion channels and Rac1 signaling to increase plasticity and promote myogenic differentiation, while compressive stress activates RhoA–ROCK signaling to decrease plasticity and enhance osteogenic differentiation. Panel 3: Inflammatory microenvironment, where low cytokine concentrations moderately increase plasticity to promote stem cell migration and immune regulation, while high cytokine concentrations reduce plasticity to prevent apoptosis and malignant transformation.

The microenvironment surrounding stem cells is not static. During organismal development, tissue repair, or pathological states, stem cells encounter diverse microenvironmental changes such as hypoxia, nutrient deprivation, altered mechanical stress, and inflammatory stimuli (Scadden, 2014) (Figure 3). To maintain their viability and perform their biological functions, stem cells initiate adaptive remodeling processes. Cellular plasticity, by sensing microenvironmental signals and adjusting its own mechanical state, serves as a central hub regulating this adaptive remodeling.

### 4.1 Cellular plasticity and adaptive remodeling of stem cells in hypoxic microenvironments

Hypoxia represents a common microenvironmental stressor for stem cells, prevalent in embryonic development, tissue repair, and tumor microenvironments (Ayla and Karahüseyinogluc, 2019). When exposed to hypoxic conditions, stem cells exhibit enhanced cellular plasticity, driving adaptive remodeling to counteract hypoxic stress.

Hypoxia activates the hypoxia-inducible factor (HIF-1α) signaling pathway within cells. HIF-α can enhance cellular plasticity by regulating the expression of Rho family GTPases, inhibiting the activity of the RhoA-ROCK pathway, and promoting actin depolymerization (Basheeruddin and Qausain, 2024). Enhanced cellular plasticity aids stem cells in adaptive adjustments. On one hand, increased flexibility facilitates stem cell deformation, enabling migration within the dense tissue microenvironment caused by hypoxia to seek regions with more abundant oxygen and nutrients (Zanotelli et al., 2021). On the other hand, enhanced plasticity activates stem cell self-renewal signaling pathways, promoting stem cell self-renewal and maintaining the stability of the stem cell population. This reserves cellular resources for subsequent tissue repair or stress responses (Aragona et al., 2013). For instance, in myocardial ischemia injury models, bone marrow-derived mesenchymal stem cells undergoing flexible enhancement under hypoxic conditions not only migrate more efficiently to ischemic myocardial tissue but also maintain cell numbers through self-renewal. Concurrently, they enhance their differentiation potential toward cardiomyocytes, thereby participating in myocardial tissue repair (Engler et al., 2008).

### 4.2 Cellular plasticity and adaptive remodeling of stem cells in response to mechanical stress changes

Mechanical stress constitutes a vital component of the stem cell microenvironment, with significant variations observed across different tissues and organs. Bone marrow stem cells endure substantial compressive stress (Thompson et al., 2012), while vascular endothelial progenitor cells are subjected to blood flow shear stress (Cunningham and Gotlieb, 2005). When the mechanical stress environment changes, stem cells achieve adaptive remodeling by adjusting their cellular plasticity to accommodate the new mechanical microenvironment.

When stem cells are subjected to tensile stress stimulation, their cellular plasticity undergoes adaptive enhancement. Tensile stress activates mechanosensitive ion channels on the cell membrane, converting mechanical signals into chemical signals. This subsequently activates the Rac1 pathway, promoting actin branching and enhancing cellular plasticity (Jin et al., 2020). This increased flexibility enables stem cells to withstand greater tensile deformation, preventing structural disruption. Furthermore, flexibility adjustments regulate the differentiation direction of stem cells. For instance, applying cyclic tensile stress to bone marrow mesenchymal stem cells enhances their flexibility, making them more inclined to differentiate into myoblasts to adapt to the mechanical environment of muscle tissue (Kurpinski et al., 2009; Shin et al., 2013). When stem cells are subjected to compressive stress, their cellular plasticity exhibits an adaptive decrease. Compressive stress activates the RhoA-ROCK signaling pathway, promoting actin polymerization and contraction, thereby increasing cellular rigidity and reducing flexibility (McNamara et al., 2009). This flexibility adjustment enhances stem cells' resistance to compressive deformation, maintaining cellular morphology and functional stability. Concurrently, compression stress-induced reduction in cellular plasticity promotes differentiation of stem cells into compressive stress-bearing tissue cells. In skeletal stem cells, decreased flexibility under compressive stress significantly enhances osteoblast differentiation potential, contributing to bone tissue repair and remodeling (Kearney et al., 2010).

### 4.3 Cellular plasticity and adaptive remodeling of stem cells in response to the inflammatory microenvironment

The inflammatory microenvironment commonly arises in pathological states such as tissue injury and infection, with inflammatory cytokines like TNF-α, IL-6, and IL-1β serving as core signaling molecules (Chen et al., 2017). When exposed to this environment, stem cells undergo adaptive alterations in cellular plasticity, thereby regulating their biological functions and achieving adaptive remodeling to participate in inflammation resolution and tissue repair.

Inflammatory cytokines influence stem cellular plasticity by regulating cytoskeletal remodeling. Low concentrations promote moderate actin depolymerization, slightly enhancing cellular plasticity (Li et al., 2017). Conversely, high concentrations of inflammatory factors cause excessive actin polymerization, reducing cellular plasticity. Within a low-concentration inflammatory microenvironment, enhanced cellular plasticity promotes stem cell migration, enabling rapid arrival at sites of inflammatory injury (Shi et al., 2012). Simultaneously, increased flexibility activates the immune regulatory functions of stem cells, promoting the secretion of anti-inflammatory factors and contributing to the resolution of inflammation.

In a high-concentration inflammatory microenvironment, reduced cellular plasticity inhibits excessive migration of stem cells, preventing apoptosis or malignant transformation caused by overexposure to inflammatory cytokines (Grivennikov et al., 2010). This flexibility reduction also enhances stem cell anti-apoptotic capacity by upregulating anti-apoptotic protein expression, thereby sustaining stem cell survival and ensuring subsequent tissue repair. In a skin injury inflammation model, low concentrations of inflammatory factors induce increased flexibility in skin stem cells, promoting their migration to the injury site and differentiation into epidermal cells to participate in wound repair (Rognoni and Watt, 2018). When the inflammatory response intensifies and inflammatory factor concentrations rise, skin stem cellular plasticity decreases, enhancing anti-apoptotic capacity to prevent cell death.

## 5 Research outlook and application prospects

Currently, research on the role of cellular plasticity in stem cell malignant transformation and adaptive remodeling has made some progress, but numerous issues remain to be addressed. At the basic research level, existing studies predominantly focus on the regulation of cellular plasticity by single signaling pathways or individual microenvironmental factors. However, the dynamic equilibrium of cellular plasticity arises from the coordinated action of multiple pathways and factors. Future research should further explore the cross-regulatory mechanisms between different signaling pathways and the patterns of cellular plasticity changes under the combined influence of multiple microenvironmental factors. Additionally, key aspects of the molecular mechanisms by which cellular plasticity regulates malignant transformation and adaptive remodeling in stem cells remain unclear. For instance, the specific molecular targets through which cellular plasticity senses microenvironmental signals require in-depth investigation using higher-resolution imaging techniques and precise molecular biology methods.

In terms of application prospects, cellular plasticity, as a key regulator of stem cell physiological and pathological functions, offers novel approaches for diagnosing and treating related diseases. In disease diagnosis, abnormal changes in cellular plasticity can serve as early diagnostic markers for malignant transformation of stem cells. For instance, detecting flexibility alterations in bone marrow mesenchymal stem cells or neural stem cells via techniques like atomic force microscopy enables early detection of malignant transformation tendencies, providing a basis for early diagnosis of hematological malignancies and neurological tumors. In disease treatment, regulating cellular plasticity can achieve therapeutic effects for stem cell-related disorders. For tumors arising from malignant transformation of stem cells, intervening in cytoskeletal remodeling signaling pathways, such as inhibiting the RhoA/ROCK pathway, can restore normal cellular plasticity levels. This suppresses proliferation, invasion, and migration of tumor stem cells, thereby achieving therapeutic outcomes. On the other hand, in the field of tissue repair, enhancing the adaptability of stem cells to the damaged microenvironment by modulating their plasticity can improve their reparative capacity. For instance, by regulating the plasticity of bone marrow-derived mesenchymal stem cells, their survival, migration, and differentiation capabilities within microenvironments such as myocardial ischemia and nerve injury can be strengthened, thereby enhancing the efficacy of stem cell transplantation therapy.

Furthermore, with advancements in biomaterial technology, it is possible to design biomaterials with specific mechanical properties to mimic the optimal mechanical microenvironment for stem cells. By regulating stem cellular plasticity through the material’s mechanical signals, this approach can guide stem cell proliferation, differentiation, and adaptive remodeling. This regulatory model linking “material mechanics-cellular plasticity-stem cell function” opens new avenues for stem cell applications in fields such as tissue engineering and regenerative medicine.

## 6 Conclusion

Cellular plasticity, serving as a vital bridge between stem cells and their microenvironment, plays a pivotal role in both malignant transformation and adaptive remodeling of stem cells. Future investigations into the regulatory mechanisms of cellular plasticity and its functions in physiological and pathological processes of stem cells may offer novel strategies for diagnosing and treating stem cell-related diseases, thereby advancing the fields of life sciences and medicine.
